# Randomised, double-blind, placebo-controlled, parallel-group, multicentric, phase IIA clinical trial for evaluating the safety, tolerability, and therapeutic efficacy of daily oral administration of NFX88 to treat neuropathic pain in individuals with spinal cord injury

**DOI:** 10.1038/s41393-024-01006-4

**Published:** 2024-06-19

**Authors:** Pablo V. Escribá, Ángel M. Gil-Agudo, Joan Vidal Samsó, Judith Sánchez-Raya, Sebastián Salvador-de la Barrera, Vanesa Soto-León, Natacha León-Álvarez, Bosco Méndez Ferrer, Miguel David Membrilla-Mesa, Carolina Redondo Galán, Jesús Benito-Penalva, Antonio Montoto-Marqués, Javier Medel Rebollo, Ramiro Palazón García, Francisco Gutiérrez Henares, Marc Miralles, Manuel Torres, Ana B. Nieto-Librero, David García Marco, Carmela Gómez, David Jimeno, Antonio Oliviero

**Affiliations:** 1 Universidad Islas Baleares, Palma, Islas Baleares Spain; 2 Hospital Nacional de Parapléjicos, SESCAM, Toledo, Spain; 3 Fundación Institut Guttmann, Institut Universitari de Neurorehabilitació adscrit a la Universitat Autònoma de Barcelona, 08916 Badalona, Spain; 4 Hospital Campus Vall d’Hebron, Barcelona, Spain; 5 Complejo Hospitalario Universitario A Coruña, A Coruña, Spain; 6 Hospital Los Madroños, Brunete, Madrid Spain; 7 Hospital Universitario Virgen del Rocio, Sevilla, Spain; 8 Instituto de Investigación Biosanitaria ibs.GRANADA, Granada, Spain; 9 Hospital Universitario Virgen de las Nieves, Granada, Spain; 10 Departamento de Estadística, Universidad de Salamanca, Salamanca, Spain; 11 Instituto de Investigación Biomédica de Salamanca, Salamanca, Spain; 12 Neurofixpharma SA, Salamanca, Spain

**Keywords:** Pain management, Quality of life

## Abstract

**Study design:**

Double-blind, randomized, placebo-controlled, parallel-group multicentric phase IIA clinical trial.

**Objective:**

To assess the safety and tolerability of oral administration of NFX-88 in subjects with chronic spinal cord injury (SCI) and explore its efficacy in pain control.

**Setting:**

A total of 7 spinal cord injury rehabilitation units in Spain.

**Methods:**

A total of 61 adult with traumatic complete or incomplete spinal cord injury (C4-T12 level), were randomised 1:1:1:1 to a placebo, NFX88 1.05 g, 2.1 g, 4.2 g/day for up to 12 weeks. The placebo or NFX-88 was administered as add-on therapy to pre-existing pregabalin (150–300 mg per day). Safety and tolerability were evaluated, and the Visual Analogue Scale (VAS) was the primary measure to explore the efficacy of NFX-88 in pain control.

**Results:**

No severe treatment-related adverse effects were reported for any of the four study groups. 44 SCI individuals completed the study and were analysed. The data obtained from the VAS analysis and the PainDETECT Questionnaire (PD-Q) suggested that the combination of NFX88 with pregabalin is more effective than pregabalin with placebo at reducing neuropathic pain (NP) in individuals with SCI and that the dose 2.10 g/day causes the most dramatic pain relief.

**Conclusions:**

NFX88 treatment was found to be highly safe and well tolerated, with the dose of 2.10 g/day being the most effective at causing pain relief. Thus, the promising efficacy of this first-in-class lipid mediator deserves further consideration in future clinical trials.

## Introduction

Spinal cord injury (SCI) is a condition derived from accidental trauma (in ~83–90% of the cases) or other conditions affecting spinal cord integrity [1, 2]. This pathology is associated with a variety of health problems, with neuropathic pain (NP) being one of the symptoms that most significantly affects the quality of life [3]. Neuropathic pain is a non-nociceptive chronic pain caused by damage or diseases that affect the somatosensory nervous system [4] and is a common clinical manifestation that affects about two-thirds of individuals after SCI [5–7]. Also, neuropathic pain following SCI may persist for many years after acute injury occurs [8–10]. This pain is often severe and refractory to treatments that can involve the use of anticonvulsants, antidepressants, analgesics, and spasticity medications [11–13]. Severe neuropathic pain is a strong predictor of reduced quality of life after SCI and can lead to mood alterations and sleep disturbances [1, 3]. In addition, neuropathic pain is a condition that is difficult to clinically manage, as it is typically resistant to therapy [6, 8, 14].

Many individuals with SCI start treatment by taking a single drug such as pregabalin or gabapentin, although additional drugs are sometimes required to reduce the associated chronic pain (first-line treatment often includes one of these two drugs plus amitriptyline or duloxetine) [12]. Pregabalin is an anticonvulsant that has been studied for its application in the management of neuropathic pain in SCI individuals [10]. Consequently, this drug has been recommended as the first-line treatment for neuropathic pain due to SCI [15]. The recommended doses for pregabalin are between 150–600 mg/day; however, most of the SCI individuals in our care were treated with 150–300 mg/day. Despite this approach, often taken together with other drugs, many SCI individuals still report experiencing moderate to severe pain [12].

In animal models, NFX88 has proven to be effective at alleviating neuropathic pain and reducing inflammation [16–18]. This hydroxylated fatty acid becomes incorporated into the plasma membrane both as a free fatty acid and also as an acyl chain of phospholipids and other acyl glycerols [19]. In addition, this bioactive lipid regulates lipid metabolism, inducing changes in the plasma membrane that cause relevant changes in the localization and activity of signalling proteins [20, 21]. This regulatory effect induces the modulation of the expression of genes involved in pathophysiological processes triggered by nerve lesions [18]. Some of these regulated genes, such as phospholipases A1 and A2 (PLA1, PLA2) and phospholipase D (PLD), contribute to the synthesis of lysophospholipids, in general, and phosphatidic acid, in particular [22]. In addition, enzymes involved in the synthesis of prostaglandins are also downregulated by NFX88, whereas genes related to neuronal proliferation and damage recovery are upregulated [18]. Thus, the combination of neuroregeneration and the prevention of inflammation could be involved in the effects mediated by NFX88. The NFX88 effects on inflammation mediators may play a role in pain reduction. In this scenario, the pro-inflammatory lipid mediator, lysophosphatidic acid, is necessary for initiating neuropathic pain following nerve injury [23]. Both lysophosphatidic acid and lysophosphatidylcholine are produced by the signals derived from nerve injury, and their synthesis is inhibited by morphine and NFX88 through different mechanisms of action [18, 24]. Moreover, inflammation mediators may play an important role in the genesis and severity of pain [25]; hence it is probable that antinflammatory effects may reduce pain severity. Taken together, the studies that have been carried out indicate that NFX88 is a first-in-class bioactive lipid with a mechanism of action that is different from the rest of the drugs used to manage pain.

Regarding the pain associated with SCI, it has been found that NFX88 is safe and effective in preclinical models, findings that still need to be confirmed in humans. Therefore, the rationale for conducting this Phase IIA clinical trial (CT) was to assess whether NFX88 would be safe and well tolerated in individuals suffering from spinal cord injury. Moreover, the hypothesized mechanism of action is different from that of other drugs commonly used to treat SCI pain. For this reason, the tolerability, safety, and preliminary effectiveness of NFX88 in relieving neuropathic pain in SCI individuals were assessed. To this end, both placebo and NFX88 were administered to participants as an add-on treatment to pregabalin (150–300 mg daily dose) at a stable dose at least one month before their inclusion in this clinical study.

The primary objective of this work was to evaluate the safety and tolerability of the use of NFX88 for 90 days to treat SCI individuals with neuropathic pain. In addition, the objective was to explore the preliminary therapeutic efficacy of a 90-day treatment with NFX88 using validated pain measurement scales. The data obtained have been reported following the CONSORT guidelines. However, our long-term objective is to use these data to design a Phase IIB/III clinical trial that should confirm the safety and efficacy of NFX88 to treat SCI-induced neuropathic pain.

## Methods

### Participants

#### Eligibility criteria for participants

The study population consisted of male and female adult individuals, aged between 18 and 80 years of age, with traumatic spinal cord injury due to complete or incomplete C4-T12 for more than three months. The participants also included those who had been diagnosed with neuropathic pain, with a pain score ≥4.0 according to the Visual Analogue Scale (VAS features a 10 cm line where individuals mark their level of pain, ranging from 0.0, indicating no pain, to 10.0 indicating maximum pain. Results were recorded in centimetres to one decimal place corresponding to a 100-point scale), during the last week before the randomisation date, and those who had been stably treated at a stable dose for at least the last month with pregabalin in the range of 150–300 mg/day, which was maintained at the same dose until the end of the study. Participants could not be treated with opiates or cannabinoids, but those who had been treated with stable doses of other neuroactive drugs (antidepressants, anticonvulsants, antispastic, and similar medicines), at a stable dose, at least during the last month, could also be recruited (this medication was kept stable). SCI individuals were excluded from the trial if they had been treated with opiates and/or cannabinoids or if they had a history of alcohol or drug abuse within 6 months before the screen, and participants who had psychiatric disorders or moderate or severe cognitive impairment were also excluded. In addition, SCI individuals were excluded if they had severe arterial hypo or hypertension (a blood pressure outside of 90–160 for systolic pressure and 50–115 for diastolic pressure), if they were pregnant or lactating, showed evidence of significant liver or kidney disease, or if they had clinically significant diseases recorded in their medical history or detected at the time of the physical examination and/or clinically significant laboratory analyses (haematology, biochemistry, and urinalysis) or ECG.

Data were collected at seven different hospitals in Spain: 1) National Hospital for Paraplegics of Toledo; 2) Vall d’Hebron University Hospital, Barcelona; 3) A Coruña University Hospital Complex; 4) Virgen de las Nieves University Hospital, Granada; 5) Institut Guttmann, Barcelona; 6) Hospital Los Madroños, Brunete, Madrid; and 7) Virgen del Rocío University Hospital, Sevilla. All centres had extensive experience in SCI management.

### Trial design

**Fig. 1:**
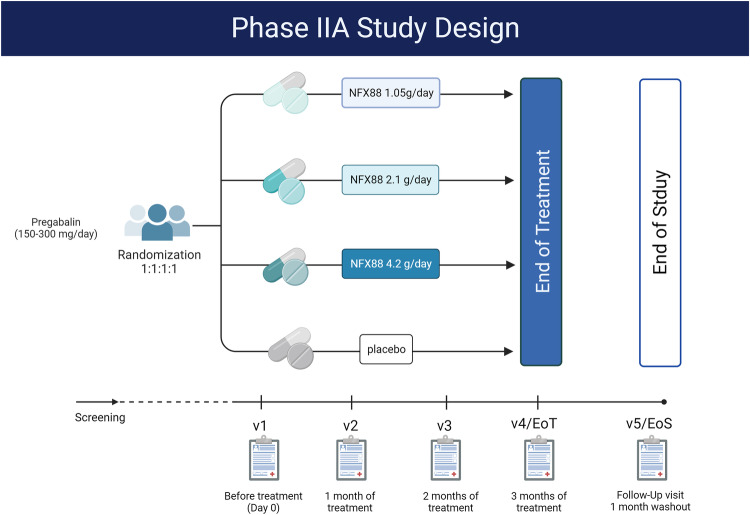
Clinical trial design. NFX88 treatment started after Visit 1 (V1). V2, V3, and V4 (EoT, end of treatment) were carried out after 1, 2, and 3 months of treatment, respectively. During the last month, participants did not receive NFX88, and a final follow-up visit (V5, EoS, end of study) was carried out to determine the effect of NFX88 withdrawal.

The design of the clinical trial is described in Fig. 1 and is a phase IIA randomised, double-blind, placebo-controlled, parallel-group, and multicentric trial, carried out between 2019 and 2022, involving individuals with neuropathic pain due to spinal cord injury (EudraCT number: 2018-004792-13/ Clinicaltrials.gov: NCT04148573). The allocation ratio was 1:1:1:1 to a placebo, NFX88 1.05 g, 2.1 g, 4.2 g/day. The treatment duration was 12 weeks, and the whole duration of the study was ~17 weeks divided as follows: a screening period (one week) including one visit (visit at screening-VS), a treatment period (12 weeks) including 4 visits (V1, V2, V3, V4/End of Treatment, EoT) and a follow-up period (4 weeks) that ended on the fifth visit (V5/End of Study, EoS).

This study was approved by the Institutional Review Board (Regional Committee of Ethics for Research with Medicines, CEIm, of the Community of Madrid: ref EC 06/19) and by the Spanish Drug Agency (AEMPS). All participants provided written informed consent before participating in the trial. This study was carried out in compliance with applicable regulations and guidelines and following the principles of the Declaration of Helsinki and the International Conference on Harmonization Good Clinical Practice guidelines. The CT was fully monitored by a professional Contract Research Organization (CRO) and all data presented here were reported after the final report had been sealed.

The study consisted of a 1-week baseline screening period, followed by the participants being randomly assigned one of the doses of NFX88 or the placebo. Each subject was administered four tablets of the study drug or the placebo three times a day over 90 days (from V1 to V4, see Fig. 1). Participants with 1.05 g/day doses received 1 tablet of 350 mg + 3 placebo tablets three times per day, 2.1 g/day group received 2 tablets of 350 mg + 2 placebo tablets, three times per day, participants allocated at 4.2 g/day group received 4 tablets of 350 mg, three times per day and the placebo group received 4 placebo tablets, 3 times per day. Visits occurred on the first day of treatment (V1), 30 days later (V2), 60 days later (V3), and 90 days later (V4/EoT). A follow-up visit occurred 4 weeks after treatment completion (V5/EoS) and only the participants who had received at least 75% of the planned treatment were included in subsequent analyses.

### Outcomes (pre-specified primary and secondary outcome measures)

**Fig. 2:**
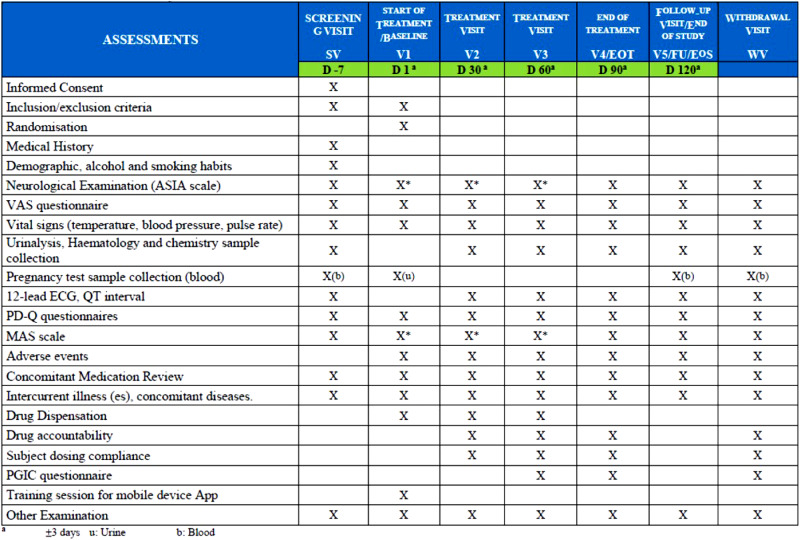
Chronogram. D: days of treatment with respect to the treatment initiation (D1, day 1). For other details, see text.

Safety and efficacy variables were collected during the study. The study chronogram is shown in Fig. 2. The outcome to analyse the safety and tolerability of NFX88 in SCI individuals was based on the incidence, severity, and causality of any adverse events (AEs); changes in vital sign parameters and the physical examination, changes in laboratory values, changes in ECGs, and changes detected through Modified Ashworth Scale (MAS) (e.g. to monitor spasticity worsening) and the International Standards for Neurological Classification of Spinal Cord Injury (ISNCSCI) (e.g. to monitor neurological worsening) scores.

The following information was recorded as efficacy variables: Visual Analogue Scale (VAS), PainDETECT Questionnaire (PD-Q) [26, 27], and Patient Global Impression of Changes (PGIC).

### Blinding

Identification of placebo versus NFX88 treatment was blind. SCI individuals were randomly assigned to receive the two drugs in a double-blind model such that neither the investigator nor the participant knew which combination was being administered. Blinding of either medication was ensured as the packaging of both NFX88 and the placebo were identical. Personnel involved in conducting the study did not have access to the randomisation code before the blind trail was officially broken. The study participants were notified through the informed consent form that they would be receiving a blind treatment; that is, they would not be informed about which drug (Investigational or Comparator) was being administered. The treatments assigned to each participant were kept blind to the study team until database lock. In the event that an individual subject had a medical emergency or became pregnant, where knowledge about the treatment being administered was critical to the person’s health, the blind for that subject was broken by the researcher. Unblinding was only granted if knowledge of the study treatment was essential for the appropriate clinical management or welfare of the participants. No unblinding occurred in this study.

#### Blinding the analytical process

The statistician was not blinded to participant allocation. To avoid bias, the analysis was carried out in several phases, where true allocation was only added during the final phase. These phases were:

Phase 1—data pre-processing and cleaning.

Phase 2—the programming of models, tables, and graphs (the statistician wrote the code for all statistical models, tables, and graphs using the data-2 dataset).

Phase 3—running the code on unblinded data (data managers provided the true allocation variable to the statistician who incorporated it into the clean data-2 dataset, which generated the data-3 dataset. The code produced in Phase 2 was run in data-3 and a report was automatically produced with the unblinded results of the analysis).

Phase 4—an unplanned exploratory efficacy analysis was performed on all unblinded data.

### Randomisation

All eligible participants fulfilling all inclusion criteria and no one of the exclusion criteria were randomly assigned to the NFX88 or placebo treatment arms in a 1:1:1:1 ratio. Due to the small number of SCI individual recruited in each group, a unique, centralized with no stratification (centres or participants), and randomised list was generated. The randomisation was done by creating blocks of multiples of 4 (so 4, 8, 12….). The size of the blocks was kept secret by the data manager until the end of the study to minimize the chance of a person involved in the study guessing the allocation of the next participant. All SCI individuals participating in this study were not allowed to be randomised in this study again.

### Statistical methods

#### Sample size calculation

As this is a Phase IIA CT, the sample size was determined to demonstrate safety. The power of this trial lies in the probability of detecting cases of AEs caused by the drug used in the treatment arms. This power depends on the true (unobservable) risks of those AEs (the higher the risk the higher the chance of detecting cases). The sample size was determined to guarantee a power of 80% for detecting any AE that has an underlying risk of 5% of happening in the clinical trial participants during the treatment (Table 1). Eleven subjects per arm, from a total of 44 participants, was estimated to be enough to evaluate safety. Based on this calculation, groupings of 15 participants per arm were planned. In Table 1, we show the sample size calculation for guaranteeing a power of 80% to detect any AE that has an underlying risk of 5% of happening in the participants during the treatment.

**Table 1:** Sample size estimation.

Sample Size		If true risk of AE is…			
Per arm	Treated	2.5%	5%	10%	15%
15	45	68%	90%	99%	99.9%
14	42	66%	88%	99%	99.9%
13	39	63%	87%	98%	99.9%
12	36	60%	84%	98%	99.7%
11	33	57%	82%	97%	99.5%
10	30	53%	79%	96%	99.2%
Sample size required		Powers to detect AE (probability of seeing at least one case of AE)			

#### Statistical methods used to compare groups for primary and secondary outcomes

##### Safety assessment (main objective)

The population used to test safety included all participants who were randomised, including those who left the study for different reasons. For this analysis, each AE was coded as a binary variable (Present/Absent) for each participant. AEs defined as “unsafe levels” of laboratory parameters or clinical outcomes (vital signs, ECGs, ISNCSCI, and MAS scales) were also coded as binary. The clinical researcher was the person to assess whether the AEs were related to the treatment or not. Several binary variables were calculated if the same AE occurred to varying degrees of severity. Tables with counts and proportions of each AE in each arm were compiled. For the intervention arms, exact confidence intervals for the proportion of each AE were estimated. A comparison of the risk of each AE between arms was done using Fisher’s Exact test. To increase power, the participants from the three intervention doses were analysed together in one intervention arm. If some AEs were found to be relatively common, a logistic regression model was built to examine if there was a dose-response effect on the probability of such an adverse event happening. A variable with the count of repetitions was created for cases where it was considered appropriate to compare AEs occurring in an individual more than once. For the intervention arms, confidence intervals for the rates were calculated and the rates between arms were compared using the Poisson regression model. Drop-out participants, those leaving the study before the follow-up visit and replaced by other individuals, were also included in the analysis of safety. In this analysis, there is a potential for bias if the total time the participant is in the trial (and the total amount of drug taken) varies considerably in each trial arm (because of differences in the number of participants and the time at which they abandoned the trial). In situations like these, a sensitivity analysis was done considering individual time within the study by using rates of AEs over person-time in the study in Poisson models.

##### Efficacy assessment (secondary objective)

The Phase IIA CT presented here was specifically focused on determining safety profiles through the use of an oral tablet formulation and on testing preliminary efficacy at different dosage levels. A group of participants was administered the drug at different doses to determine the recommended dose for phase 2B and 3 clinical studies. In this trial, the power analysis was focused on determining safety, therefore, assessing efficacy should be considered exploratory. The efficacy analysis included all participants who were randomised, had at least 75% of treatment compliance and had completed the appropriate VAS, PD-Q, or PGIC questionnaires. The variable VAS used to analyse efficacy was coded as a continuous non-parametric variable. PD-Q was coded as being continuous (from 0 to 38) but also as a categorical variable with three possible categories (10). The three possible categories were low, intermediate/uncertain, and high probability of neuropathic component of the pain. PGIC was analysed as a categorical variable recoded (due to the small sample size) in three categories: improved (a score between 1 and 3 values), no change (a score of 4), and worse (score between 5 and 7).

The preplanned/predeclared first exploratory efficacy endpoint was the change in VAS (principal efficacy variable) from baseline to V4/EoT. The difference between V1 and V4/EoT, in the different groups, was analysed using the Kruskal–Wallis test. Due to the exploratory nature of the Phase IIA CT, we included additional analyses to the statistical plan after analysing the results.

Further analyses of the VAS were performed, using a Friedman test (Conover’s Test was included for post hoc analysis), on the four groups (doses) that were separately analysed to evaluate the time course of the effects of NFX88. After a visual inspection of the data, three-time points were included in the Friedman test (V1, V3, and V4/EoT). Due to the exploratory nature of the analysis, the same data obtained for the four groups were also compared directly using the Wilcoxon Test (V1 vs. V3, V1 vs. V4/EoT). Our a priori hypothesis was that NFX88 would reduce neuropathic pain, so when we compared V1 vs. V3 and V1 vs. V4/EoT, we reported the p-value of the post hoc analysis considering the hypothesis of V1 different from V3 and V4/EoT (two tails) and V1 higher than V3 and V4/EoT (one tail). For all the analyses, the exact p-value is indicated to provide a reference for the robustness of these exploratory analyses.

To further explore the efficacy at different doses, the number of responders at V4/EoT using different definitions was reported. Due to the exploratory nature of our efficacy analyses, we defined the responders in four different ways: 1) participants with a ≥ 30% reduction in pain (based on VAS); 2) participants with a ≥ 50% reduction in pain (based on VAS); 3) participants with a ≥ 1.5-point reduction in pain according to the VAS; and 4) participants with a ≥ 2-point reduction in pain according to the VAS. These categorizations (≥30%, ≥50%, ≥1.5-point, and 2-point reduction in pain according to the VAS) were arbitrarily decided to allow data presentation in individuals with more or less intense pain relief. Moreover, we descriptively report the number of drop-out participants which can be considered a measure of treatment failure (being determined by AEs, treatment discomfort, and lack of efficacy) and the PGIC for all groups. These variables (responders vs. non-responders, drop-outs vs. participants that completed the treatment, and PGIC) were separately analysed using Chi-squared analysis.

We explored the use of PD-Q (secondary efficacy variable) to test NFX88 analgesic effects in two different ways: 1) the score (assuming that the higher the score the more intense is the neuropathic pain); 2) the change in the neuropathic component of pain. The differences found in the PD-Q scores between V1 and V4/EoT were analysed using a Kruskal-Wallis test. This is an exploratory valuation as PD-Q is not the appropriate scale to test pain severity. The PD-Q was also analysed as a categorical variable with the following cut-offs: a score of ≤ 12 indicates that pain is unlikely to have a neuropathic component, a score of ≥ 19 suggests that pain is likely to have a neuropathic component, and a score between these values (13–18) indicates that the result is uncertain. The main idea with this analysis is that NFX88 is theoretically useful only for neuropathic pain. So, if the number of individuals with pain that is likely to have a neuropathic component decrease, we can indirectly consider that the drug is effective on reducing at least this component of pain.

##### Stratification based on the initial PD-Q classification

The PD-Q quantifies the probability that the pain described by the participants is neuropathic in origin. According to its mechanism of action, NFX88 should be more efficient in treating this type of participants. Therefore, we explored its efficacy in a subgroup of participants where the probability of experiencing pain with a neuropathic component was greater than 90% (PD-Q ≥ 19). In this subgroup, the difference between the VAS scores obtained at the end-of-treatment visit (V4) and the first visit (V1) for the four treatment arms (placebo, NFX88 1.05 g/day; NFX88 2.1 g/day NFX88 4.2 g/day), the time course of the four treatment arms, the quantification of responders and the PGIC were determined.

## Results

### Subject demographics and characteristics

The assessment of the safety of NFX88 included all participants who were randomised, including those who had left the study. In addition, an exploratory analysis of efficacy was performed and only those participants completing the study were analysed.

**Fig. 3:**
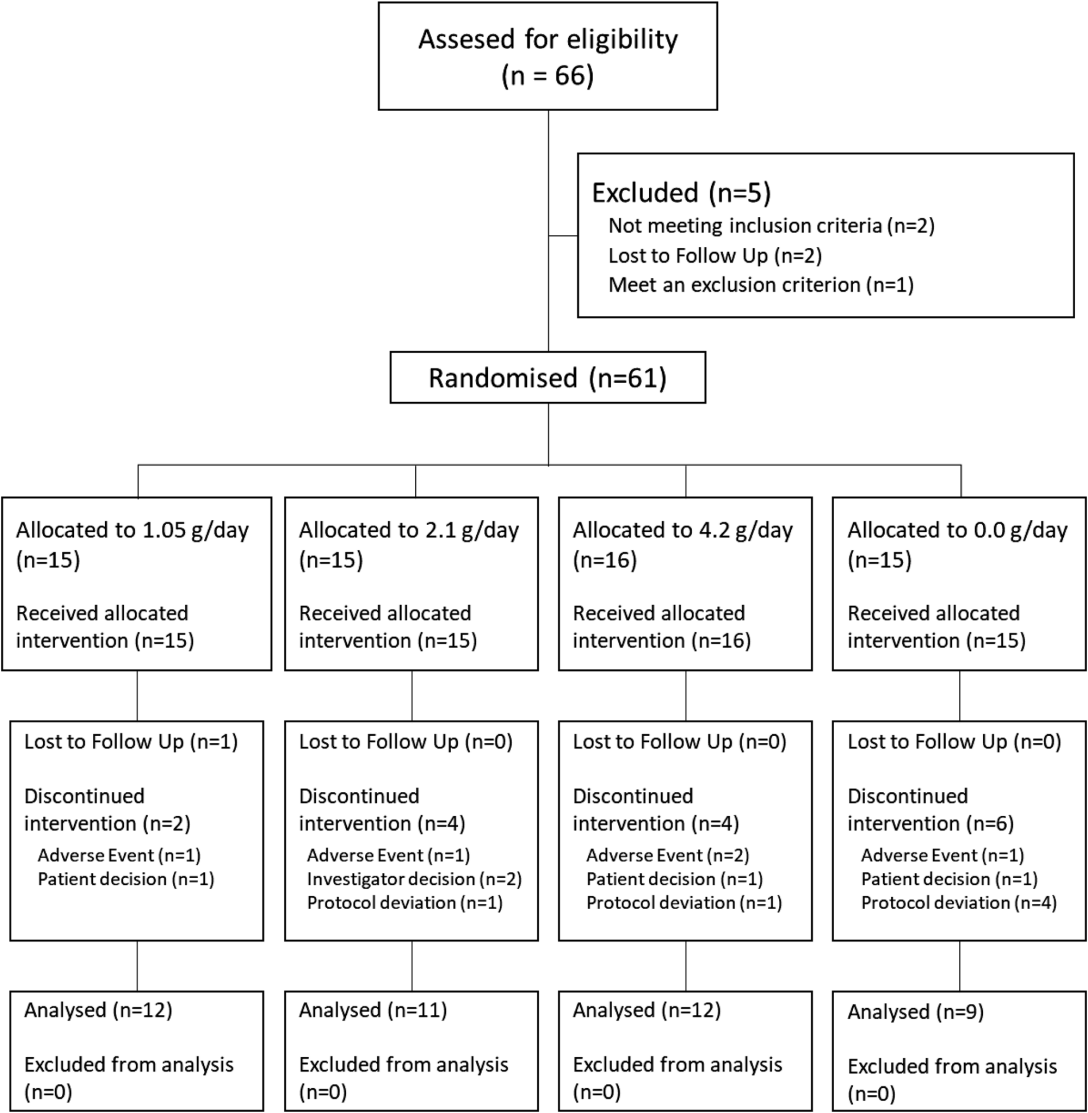
CONSORT flow diagram. The diagram shows the disposition of all subjects included in the study.

A total of 61 participants were randomised to placebo (n = 15), NFX88 1.05 g/day (n = 15), 2.1 g/day (n = 15), and 4.2 g/day (n = 16) (Fig. 3), of which 44 participants (72%) completed the study. The median exposure to treatment for the placebo group was 72 days and 87.5 days for NFX88-treated participants.

### Outcomes

#### Safety and tolerability

The demographic and baseline characteristics of the randomized participants were comparable across the treatment arms (Table 2). Of the 61 participants randomized in the trial, 46 received at least 1 dose of NFX88 (15 received 1.05 g/day, 15 received 2.10 g/day, and 16 received 4.20 g/day).

**Table 2:** Patient demographic and clinical characteristics at time of screening (n = 61).

	0.00 g/day	1.05 g/day	2.10 g/day	4.20 g/day	NFX88
	(N = 15)	(N = 15)	(N = 15)	(N = 16)	(N = 46)
Age					
Mean (SD)	47.4 (13.4)	51.0 (12.8)	48.7 (10.8)	41.7 (11.6)	47 (12.17)
Gender					
Female	6 (40.0%)	1 (6.7%)	3 (20.0%)	2 (12.5%)	6 (13%)
Male	9 (60.0%)	14 (93.3%)	12 (80.0%)	14 (87.5%)	40 (87%)
Ethnicity					
Caucasian	15 (100%)	13 (86.7%)	14 (93.3%)	16 (100%)	43 (93.48%)
Black	0 (0%)	1 (6.7%)	0 (0%)	0 (0%)	1 (2.17%)
Asian	0 (0%)	0 (0%)	0 (0%)	0 (0%)	0 (0%)
Hispanic	0 (0%)	1 (6.7%)	1 (6.7%)	0 (0%)	2 (4.35%)
Other	0 (0%)	0 (0%)	0 (0%)	0 (0%)	0 (0%)
Weight					
Mean (SD)	74.0 (15.3)	82.0 (12.1)	78.2 (11.8)	81.0 (11.6)	80.45 (11.7)
Years since lesion started					
Mean (SD)	9.93 (12.87)	9.57 (10.31)	8.78 (9.24)	6.26 (5.93)	8.16 (8.57)
Years since illness started					
Mean (SD)	9.47 (13.1)	5.67 (8.53)	5.93 (9.83)	5.76 (5.94)	5.78 (7.98)
Neurological level					
Paraplegia	8 (53.33%)	11 (73.33%)	11 (73.33)	12 (75%)	34 (74%)
Tetraplegia	7 (46.67%)	4 (26.67)	4 (26.67%)	4 (25%)	12 (26%)
AIS score					
A: Complete	7 (46.67%)	11 (73..33%)	8 (53.33%)	11 (68.75%)	30 (65.2%)
B: Incomplete	1 (6.67%)	0 (0%)	1 (6.67%)	1 (6.25%)	2 (4.34%)
C: Incomplete	0 (0%)	0 (0%)	2 (13.33%)	1 (6.25%)	3 (6.5%)
D: Incomplete	7 (46.67%)	4 (26.67%)	4 (26.67%)	3 (18.75%)	12 (26.1%)
E: Normal	0 (0%)	0 (0%)	0 (0%)	0 (0%)	0 (0%)
VAS					
Mean (SD)	6.96 (1.48)	7.02 (1.54)	6.38 (1.91)	6.86 (1.52)	6.76 (1.65)
Median [Min, Max]	6.7 [4.00, 9.60]	7.00 [4.50, 10.00]	6.50 [4.00, 10.0]	7.00 [4.4, 9.3]	7.00 [4.0, 10.0]
PDQF					
Mean (SD)	18.2 (4.51)	20.93 (6.40)	15.07 (7.44)	19.44 (7.73)	18.5 (7.49)
Median [Min, Max]	18.0 [11.00, 27.0]	21.0 [11.00, 30.0]	12.0 [6.00, 35.0]	19.0 [6.00, 31.0]	18.0 [6.00, 35.0]
Low probability	2 (13.33%)	1 (6.67%)	8 (53.33%)	4 (25%)	13 (28.26%)
Intermediate probability	6 (40%)	6 (40%)	2 (13.33%)	4 (25%)	12 (26.08%)
High probability	7 (46.67%)	8 (53.3%)	5 (33.33%)	8 (50%)	21 (45.65%)

*AIS* Asia impairment scale, *PDQF* painDETECT questionnaire final score.

**Fig. 4:**
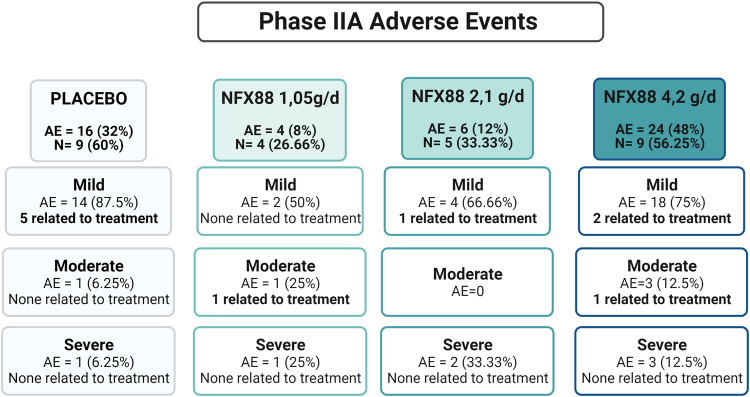
Distribution of adverse events. AE: Number of adverse events; N = Number of participants reporting the AE; Placebo: 15 participants; 1.05 g/day: 15 participants; 2.10 g/day: 15 participants; 4.20 g/day: 16 participants.

Of these 46 participants, 18 (39.1%) experienced a total of 34 adverse events, 5 of which were deemed related to the treatment (3 mild and 2 moderate). Seventy-six percent of the AEs were mild, 10% were moderate, and 14% were severe. The most common AEs were urinary infections (22%) and gastrointestinal distress (12%). Seven serious adverse events (SAEs) were reported during the CT, one of which occurred before the treatment had begun. No treatment-related SAEs were reported for any of the four study groups, but related AEs appeared at a higher rate in the placebo group than in the verum groups (5 in the placebo group vs. 5 among all three NFX88 groups). Data related to safety are summarized in Fig. 4. Of the 10 AEs related to treatment by investigators, 6 were related to gastrointestinal distress, 2 to a mild increase of transaminases, 1 was mild hypotension, and 1 was mild colitis. All AEs related to treatment were resolved without sequelae, except for one individual who still had high levels of hepatic enzymes by the end of the study. There were no statistically significant differences between the treatment and placebo groups concerning safety outcomes. Altogether, the present results indicate that all three NFX88 doses are safe and do not put the health or life quality of individuals with spinal cord injury at risk.

Fig. 4. Distribution of Adverse Events. AE: Number of adverse events; N = Number of participants reporting the AE; Placebo: 15 participants; 1.05 g/day: 15 participants; 2.10 g/day: 15 participants; 4.20 g/day: 16 participants

#### Efficacy

In the analysis of efficacy, the demographic and baseline characteristics of the participants were comparable across the three treatment arms and the placebo group (Table 3).

**Table 3:** Patient demographic and clinical characteristics at baseline (patients analyzed in efficacy analysis, n = 44).

	0.00 g/day	1.05 g/day	2.10 g/day	4.20 g/day	NFX88
	(N = 9)	(N = 12)	(N = 11)	(N = 12)	(N = 35)
Age					
Mean (SD)	47.2 (11.8)	49.8 (12.2)	49.27 (11.62)	44.92 (11.37)	47.97 (11.61)
Gender					
Female	2 (22.22%)	1 (8.33%)	1 (9.09%)	2 (16.67%)	4 (11.43%)
Male	7 (77.78%)	11 (91.67%)	10 (90.91%)	10 (83.33%)	31 (88.57%)
Ethnicity					
Caucasian	9 (100%)	12 (100%)	11 (100%)	12 (100%)	35 (100%)
Weight					
Mean (SD)	70.75 (13.63)	79.92 (11.36)	80.77 (11.98)	81.58 (13.28)	80.76 (11.9)
Years since lesion started					
Mean (SD)	13.78 (15.29)	9.28 (10.94)	9.46 (9.96)	6.60 (6.34)	8.42 (9.09)
Years since illness started					
Mean (SD)	13.08 (15.85)	5.24 (8.66)	7.30 (11.25)	5.92 (6.38)	6.09 (8.59)
Neurological level					
Paraplegia	5 (55.56%)	10 (83.33%)	8 (72.73%)	10 (83.33%)	28 (80%)
Tetraplegia	4 (44.44%)	2 (16.67%)	3 (27.27%)	2 (16.67%)	7 (20%)
AIS score					
A: Complete	5 (55.56%)	10 (83.33%)	5 (45.45%)	8 (66,67%)	23 (65,71%)
B: Incomplete	1 (11.11%)	0 (0%)	1 (9.09%)	1 (8,33%)	2 (5,71%)
C: Incomplete	0 (0%)	0 (0%)	2 (18,18%)	1 (8,33%)	3 (8,57%)
D: Incomplete	3 (33.33%)	2 (16.67%)	3 (27.27%)	2 (16.67%)	7 (20%)
E: Normal	0 (0%)	0 (0%)	0 (0%)	0 (0%)	0 (0%)
VAS					
Mean (SD)	7.27 (1.5)	7.19 (1.62)	6.56 (1.85)	6.79 (1.52)	6.85 (1.63)
Median [Min, Max]	6.7 [5.00, 9.60]	7.42 [4.50, 10.00]	6.66 [4.00, 10.0]	6.75 [4.4, 8.7]	7.00 [4.0, 10.0]
PDQF					
Mean (SD)	18.33 (4.74)	22.42 (6.29)	16.91 (7.96)	21.08 (7.5)	20.23 (7.42)
Median [Min, Max]	17.0 [12.00, 27.0]	23.5 [11.00, 30.0]	17.0 [6.00, 35.0]	21.5 [8.00, 31.0]	20.0 [6.00, 35.0]
Low probability	1 (11.11%)	1 (8.3%)	4 (36%)	2 (16.67%)	7 (20%)
Interm. probability	4 (44.44%)	3 (25%)	2 (18.18%)	3 (25%)	8 (22.86%)
High probability	4 (44.44%)	8 (66.67%)	5 (45.45%)	7 (58.34%)	20 (57.14%)

*AIS* Asia impairment scale, *PDQF* painDETECT questionnaire final score.

### Preplanned analysis (VAS, PD-Q and PGIC)

**Fig. 5:**
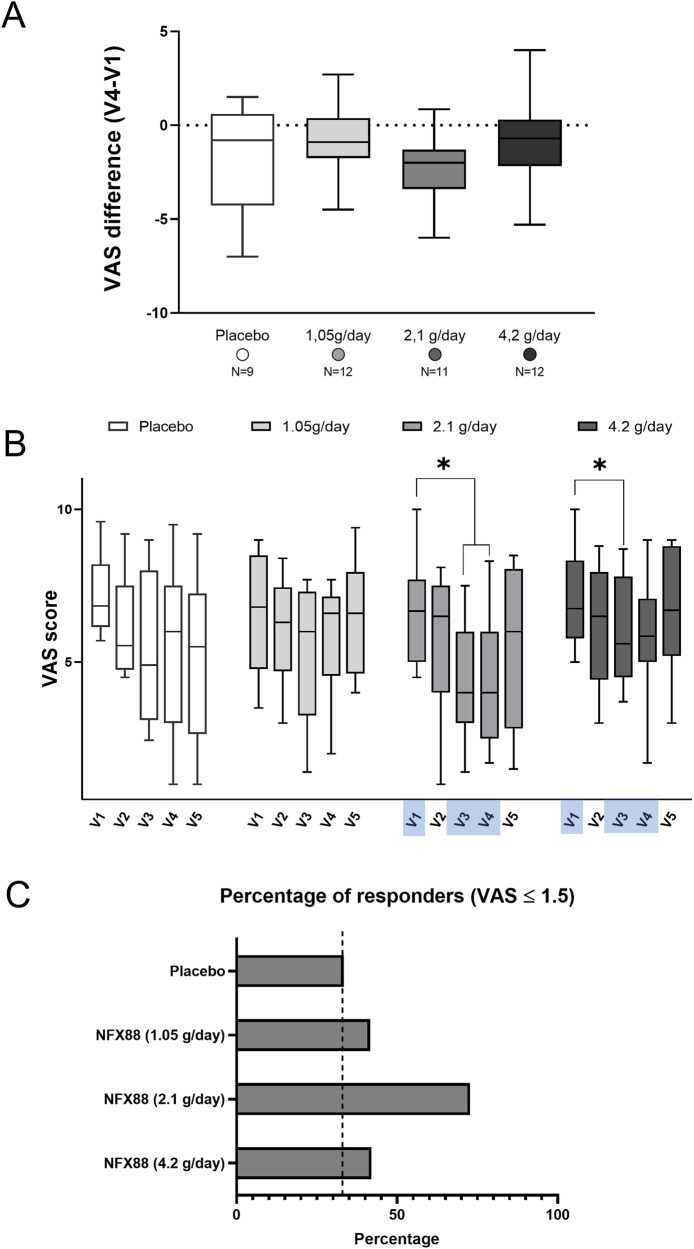
VAS scores in SCI individuals. **A** Difference in VAS score between the V4/EoT vs the V1 of the four treated arms. **B** Time course of VAS scores in different groups of participants (V1, V3, and V4/EoT included in the analysis). Statistically, differences were detected in NFX88 2.1 g/day and 4.2 g/day when time-comparisons were performed, whereas neither placebo nor NFX88 1.05 g/day participants reported any statistically difference. *p < 0.05. **C** Percentage of participants with a decrease in VAS ≥ 1.5 from V1 to V4/EoT. Note that participants treated with NFX88 2.1 g/day revealed higher pain relief in all analyses performed.

All data gathered during V1 and V4/EoT are reported in Table 4. For the analysis using the VAS, the differences found between V4/EoT and V1, for the entire sample population, are shown in Fig. 5A and the Kruskal-Wallis test revealed no significant differences (p > 0.2). When using the PD-Q, no significant differences were detected between V4/EoT and V1 (Kruskal–Wallis Test: p > 0.2). However, visual inspection of the data suggests that the analgesic effects were greater in the 2.1 g/day group. Moreover, no significant differences were found between the placebo group and the treatment arms (even when considering only the 2.1 g/day group, Mann–Whitney Test: 1 tail, p = 0.147). The median of V4/EoT-V1 differences were −0.80 for the placebo group and −2.0 for the 2.1 g/day group. These values suggested a possible (to be confirmed in further trials) greater pain reduction in the 2.1 g/day group than in the placebo-treated group of participants.

**Table 4:** Summary of exploratory efficacy outcomes.

VAS score	N°	Visit 1		Visit 4	
Treatment		Mean (SD)	Median [min, max]	Mean (SD)	Median [min, max]
Placebo	9	7.17 (1.33)	6.84 [5.7, 9.6]	5.5 (2.78)	6.0 [1, 9.5]
NFX88 (1.05 g/day)	12	6.62 (1.93)	6.8 [3.5, 9]	5.92 (1.71)	6.6 [2, 7.7]
NFX88 (2.1 g/day)	11	6.63 (1.66)	6.67 [4.5, 10]	4.32 (2.25)	4.0 [1.7, 8.3]
NFX88 (4.2 g/day)	12	7.02 (1.57)	6.75 [5,10]	6.01 (1.93)	5.85 [1.7, 9]

PDQ-F	N°	Visit 1		Visit 4	
Treatment		Mean (SD)	Median [min, max]	Mean (SD)	Median [min, max]
Placebo	9	18.89 (6.33)	20 [10,28]	12.67 (7.09)	13 [3,24]
NFX88 (1.05 g/day)	12	21 (5.73)	22 [11,31]	19.08 (5.14)	21 [11,26]
NFX88 (2.1 g/day)	11	16 (7,24)	15 [9,35]	10.82 (7.51)	9 [1,28]
NFX88 (4.2 g/day)	12	22 (7.87)	23 [8,32]	19.08 (7.16)	20 [9,31]

PDQ-F patients^a^	N°	Screening Visit		Visit 4	
Treatment		Low/Inter (n, %)	High (n, %)	Low/Inter (n, %)	High (n, %)
Placebo	9	5 (55.56%)	4 (44%)	7 (77.78%)	2 (22.22%)
NFX88 (1.05 g/day)	12	4 (33.33%)	8 (66.67%)	5 (41.67%)	7 (58.33%)
NFX88 (2.1 g/day)	11	6 (54.54%)	5 (45.46%)	10 (90.9%)	1 (9.1%)
NFX88 (4.2 g/day)	12	5 (41.67%)	7 (58.33%)	5 (41.67%)	7 (58.33%)

PGIC		Improved (n, %)	No change (n, %)	Worse (n, %)	
Treatment					
Placebo		5 (55.5%)	4 (44.5%)	0 (0.0%)	
NFX88 (1.05 g/day)		7 (58.3%)	4 (33.3%)	1 (8.3%)	
NFX88 (2.1 g/day)		8 (72.7%)	2 (12.2%)	1 (9%)	
NFX88 (4.2 g/day)		5 (41.6%)	7 (58.3%)	0 (0%)	

Pain responders^b^	Drop-outs (n,%)	≥30 (n, %)	≥50% (n, %)	≥1.5 (n, %)	≥2 (n, %)
Treatment					
Placebo	6 (40%)	3 (33%)	2 (22%)	3 (33.33%)	3 (33.33%)
NFX88 (1.05 g/day)	3 (20%)	1 (8.3%)	1 (8.3%)	5 (41.6%)	2 (16.67%)
NFX88 (2.1 g/day)	4 (26.67%)	5 (45%)	4 (36%)	8 (72.72%)	6 (54.5%)
NFX88 (4.2 g/day)	4 (25%)	2 (16.67%)	2 (16.67%)	5 (41.67%)	4 (33.33%)

^a^Number of patients grouped according to their probability of neuropathic component: Low/Inter refers those patients with PDQF scores from 1 to 18. High probability included patients with PDQF scores ≥19.

^b^Responders were defined as patients showing at least 30%, 50%, 1.5- or 2-point reduction in VAS scale in the EoT visit compared to the baseline (V1).

Alternatively, the proportion of SCI individuals with a high probability of neuropathic pain decreased more between VS and V4/EoT (from 45.5% to 9.1%) in the NFX88 2.1 g/day arm (Table 4) than in any other arm. Also, the results obtained from the PGIC questionnaire indicated that more individuals in the 2.1 g/day group (72.7%, Table 4) reported a general improvement at V4/EoT.

### Time course of the four treatment arms (VAS)

The time course of the four treatment arms is shown in Fig. 5B. Visual inspection of the mean VAS values may suggest there is a greater reduction in pain in the NFX88 arms at V3 and V4/EoT (e.g. after two and three months of treatment). The Friedman test (V1, V3, and V4/EoT) showed that NFX88 significantly led to lower VAS scores over time for the 2.1 g/day group (p = 0.009) and in the 4.2 g/day group of participants (p = 0.021).

Concerning the 2.1 g/day group of participants, Conover’s Post Hoc revealed significant differences between V1 and V3 (p = 0.026) and between V1 and V4/EoT (p = 0.010). Also, in the 4.2 g/day group, Conover’s Post Hoc showed a significant difference between V1 and V3 (p = 0.012) and a non-significant difference between V1 and V4/EoT (p = 0.075).

The Wilcoxon test showed that the pain measured using the VAS was significantly reduced at two and three months after treatment with NFX88 2.1 g/day (V1 vs V3: 2 tails, p = 0.005; 1 tail, p = 0.002; and V1 vs V4: 2 tails, p = 0.003; 1 tail, p = 0.001) and 4.2 g/day group (V1 vs V3: 2 tails, p = 0.006; 1 tail, p = 0.003; and V1 vs V4: 2 tails, p = 0.154; 1 tail, p = 0.077). Also, the median score for NFX88 2.1 g/day dose was two points lower, suggesting that the large effect of this dose on reducing pain should be the subject of future CTs (Rank Biserial correlation=0.939).

### Responders of the four treatment arms (VAS)

In addition to greater pain relief, the 2.1 g/day group had the highest percentage of responders (see Table 4). The chi-square showed a tendency when we consider responders the individuals showing a reduction of at least 1.5 VAS points at V4/EoT compared to V1 (placebo against 2.1 g/day: p = 0.078).

### Dropouts’ analysis

The number of dropouts was higher in the placebo group than in the treatment arms. Of the 17 participants (out of 61) who left the study, 6/15 (40% drop-out rate) were in the placebo group, and 11/46 (24% drop-out rate) were in NFX-88 treated groups. These values, even though they are not significant (chi-square: p = 0.227) may suggest that individuals with verum treatment were more prone to continue the drug assumption.

### Stratification based on initial PD-Q classification

**Fig. 6:**
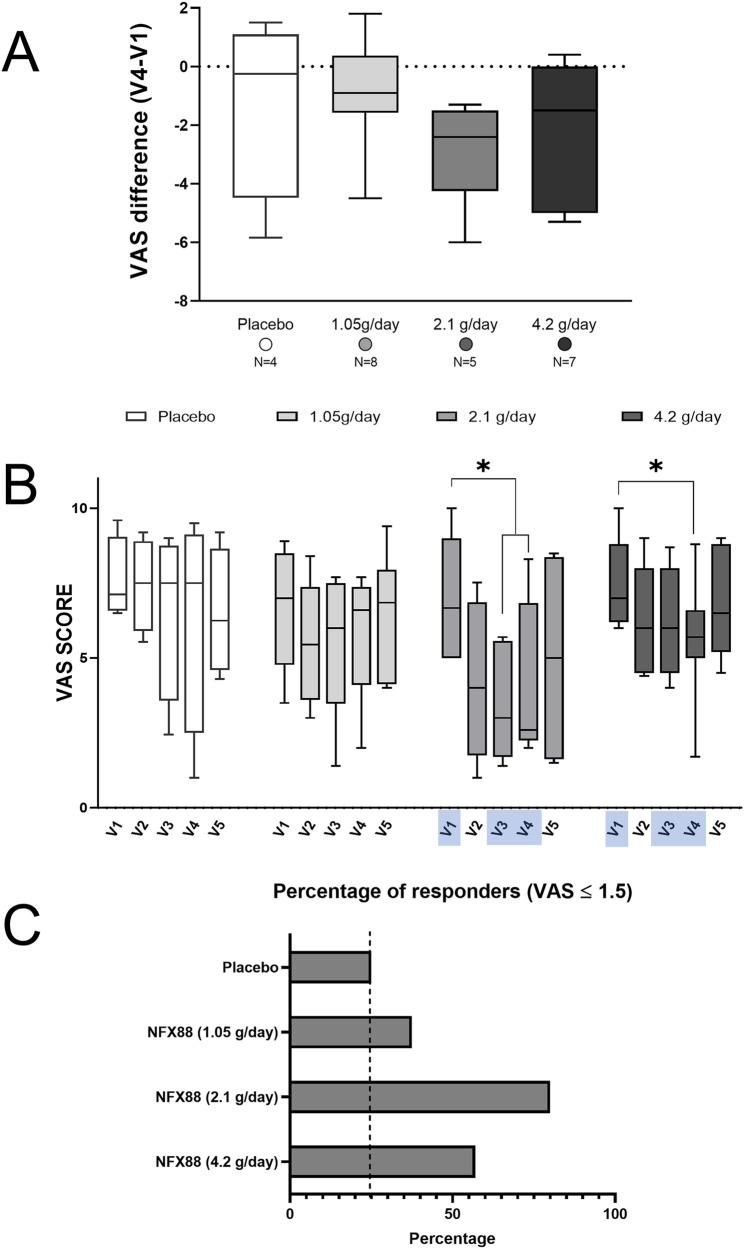
VAS scores in participants with a high probability of having neuropathic component according to the PD-Q test at VS. **A** Difference in VAS score between V4/EoT and V1 in the four treated groups. **B** Time course of the VAS scores in the different groups of participants with high neuropathic component (V1, V3, and V4/EoT included in the analysis). Statistically differences were detected in NFX88 2.1 g/day and 4.2 g/day when time comparisons were performed, whereas neither placebo nor NFX88 1.05 g/day participants reported any statistically difference. *p < 0.05. **C**. Percentage of participants with high neuropathic component that felt a decrease in VAS score ≥ 1.5, from V1 to V4/EoT.

When the data from participants with a high probability of neuropathic component were analysed, pain relief in individuals treated with NFX88 2.1 g/day and NFX88 4.2 g/day was further confirmed (Table 5, Fig. 6A). In fact, although the Kruskal-Wallis test did not show significant differences among the groups (p > 0.2), the median values of the V4/EoT-V1 differences were considerably higher in the NFX88 2.1 g/day and NFX88 4.2 g/day groups than in the placebo group (Placebo −0.25), NFX88 1.05 g/day (−0.90), NFX88 2.1 g/day (−2.40), NFX88 4.2 g/day (−1.50).

**Table 5:** Summary of exploratory efficacy outcomes.

VAS score	N°	Visit 1		Visit 4	
Treatment		Mean (SD)	Median [min, max]	Mean (SD)	Median [min, max]
Placebo	4	7.58 (1.39)	7.12 [6.5, 9.6]	6.37 (3.73)	7.5 [1, 9.5]
NFX88 (1.05 g/day)	8	6.61 (2.02)	7 [3.5, 8.9]	5.72 (2.03)	6.6 [2, 7.7]
NFX88 (2.1 g/day)	5	6.93 (2.13)	6.67 [5,10]	4.15 (2.67)	2.6 [2, 8.3]
NFX88 (4.2 g/day)	7	7.56 (1.52)	7 [6,10]	5.54 (2.13)	5.7 [1.7, 8.8]

PDQ-F	N°	Visit 1		Visit 4	
Treatment		Mean (SD)	Median [min, max]	Mean (SD)	Median [min, max]
Placebo	4	24.50 (3.11)	25 [21,28]	17 (7.75)	19 [6,24]
NFX88 (1.05 g/day)	8	24.50 (3.66)	25 [20,31]	20.88 (4.70)	23 [12,26]
NFX88 (2.1 g/day)	5	21.4 (8.02)	20 [15,35]	13.8 (9.42)	12 [3,28]
NFX88 (4.2 g/day)	7	27.14 (4.14)	28 [20,32]	22.14 (7.13)	23 [11,31]

PGIC		Improved (n, %)	No change (n, %)	Worse (n, %)	
Treatment					
Placebo		1 (25%)	3 (75%)	0 (0%)	
NFX88 (1.05 g/day)		5 (62.5%)	3 (37.5%)	0 (0%)	
NFX88 (2.1 g/day)		4 (80%)	1 (20%)	0 (0%)	
NFX88 (4.2 g/day)		3 (43%)	4 (57%)	0 (0%)	

Pain responders^a^	Drop-outs (n,%)	≥30 (n, %)	≥50% (n, %)	≥1.5 (n, %)	≥2 (n, %)
Treatment					
Placebo	3 (42.86%)	1 (25%)	1 (25%)	1 (25%)	1 (25%)
NFX88 (1.05 g/day)	0 (0%)	1 (12.5%)	1 (12.5%)	3 (37.5%)	1 (12.5%)
NFX88 (2.1 g/day)	0 (0%)	3 (60%)	2 (40%)	4 (80%)	3 (60%)
NFX88 (4.2 g/day)	1 (12.5%)	2 (28.57%)	2 (28.57%)	4 (57%)	3 (43%)

Patients with a high probability of a neuropathic component.

Drop-outs were defined as patients with a high probability of a neuropathic component who did not complete the treatment.

^a^Responders were defined as patients showing at least 30%, 50%, 1.5- or 2-point reduction in VAS scale in the EoT visit compared to the baseline (V1).

The time course of the four treatment arms, shown in Fig. 6B, was analysed including V1, V3, and V4/EoT data to be consistent with previous analyses. The Friedman test showed that NFX88 administration was associated with significantly lower VAS values over time in the 2.1 g/day (p = 0.019) and in the 4.2 g/day groups (p = 0.042). The Conover’s Post Hoc showed significant differences between V1 and V3 (p = 0.040) and between V1 and V4/EoT (p = 0.040) in the 2.1 g/day group. In the 4.2 g/day group, Conover’s Post Hoc showed a significant difference between V1 and V4 (p = 0.037) and a non-significant difference between V1 and V3 (p = 0.063).

The Wilcoxon test showed that the reduction in pain measured using the VAS was significant at two and three months after the treatment with NFX88 2.1 g/day started (V1 vs V3: 2 tails, p = 0.063; 1 tail, p = 0.031; and V1 vs V4: 2 tails, p = 0.063; 1 tail, p = 0.031) and 4.2 g/day group (V1 vs V3: 2 tails, p = 0.036; 1 tail, p = 0.018; and V1 vs V4: 2 tails, p = 0.059; 1 tail, p = 0.030). A reduction of over 2 points of the median value of the V4/EoT-V1 difference in the NFX88 2.1 g/day group shows that this drug has a potential marked effect against neuropathic pain in individuals with SCI. A finding that should be confirmed in future clinical trials (Rank Biserial correlation = 1).

According to the PGIC scale and the responders, the analyses showed great differences between NFX88-treated individuals and those receiving the placebo, with the 2.1 g/day dose being the most effective. In total, 80% of the participants treated with NFX88 2.1 g/day reported an improvement in the PGIC scale, whereas only one (25%) of the participants treated with the placebo reported any type of improvement.

## Discussion

This Phase IIA clinical trial was designed primarily to investigate the safety and tolerability profiles of NFX88 in individuals with SCI suffering from neuropathic pain; the secondary objective was to explore efficacy. The study population consisted of male and female adults, between 18 and 80 years of age, with traumatic SCI and pain. All participants had stable treatment for at least one month before participating in the study and all received pregabalin in the range of 150 to 300 mg/day. The dose used for each individual was maintained throughout the entire study. Stable doses of other neuroactive drugs, such as antidepressants, anticonvulsants, antispastic, and similar medications, were allowed and their doses were kept stable. The range of 150 to 300 mg/day of pregabalin was chosen because in the literature and our environment (12), this is a commonly used dosage.

In previous studies in animals (Wistar rats), oral 2-hydroxyoleic acid (active compound of NFX88) inhibits reflex hypersensitivity and open-field-induced anxiety after spared nerve injury at a dose of 400 mg/kg [17]. According to the dose equivalence described by Nair and Jacob [28] and previously described by the FDA [29], this dose would correspond to an oral dose of ~3.87 g per day in humans. Thus, the daily doses selected in our clinical trial to test the safety and to explore the efficacy of this compound against neuropathic pain in humans were 1.05, 2.1, and 4.2 g/day.

Treatment-related AEs in this study were consistent with an extremely high safety profile for NFX88. However, gastrointestinal symptoms were the most frequently reported AEs (both in placebo and active treatment arms) and were mild to moderate in intensity; only 1 participant abandoned the study due to this particular AE. Drop-out occurred more frequently in the placebo group of participants than in the NFX88-treated groups. The sample size was chosen to detect common side effects (with a predicted frequency of 5% or more). However, less frequent side effects should be considered in future clinical studies with a larger number of participants.

The chosen sample size was underpowered to demonstrate efficacy; therefore, we must consider the results for efficacy to be only exploratory. Visual inspection of the data may suggest that higher analgesic effects are present in the 2.1 g/day group. However, a significant difference between the V1-V4/EoT values for the placebo group and the treatment arms (even considering only the 2.1 g/day group) was not found. It is important to underline that this study was powered to test safety. Thus, there are a number of arguments suggesting that with an appropriate sample size estimation to test efficacy (that will be planned in future CTs) the data may provide evidence of it.

When considering the entire group of participants completing the study, the main findings that support efficacy are: 1) NFX88 significantly reduced VAS scores over time in the 2.1 g/day group (p = 0.009) and in the 4.2 g/day group (p = 0.021); 2), the proportion of SCI individuals with a high probability of experiencing neuropathic pain decreased in the NFX88 2.1 g/day arm (from 45.5% to 9.1%) compared to the placebo arm (from 44% to 22.2%) (Table 4); 3) the results of the PGIC questionnaire showed more individuals referring improvement in the 2.1 g/day group (72.7%, Table 4) that reported improved pain relief at V4/EoT; 4) in the 2.1 g/day group, a 2-point reduction suggested this dose had the potential to have a large effect on pain; 5) the 2.1 g/day group had the highest number of responders; and 6) the number of drop-outs was higher in the placebo group than in the treatment arms, suggesting that individuals with verum treatment were more prone to continue the drug assumption.

Therefore, NFX88 could be a potential drug for managing neuropathic pain, as it is specifically designed to directly treat the pathologic changes causing neuropathic pain after spinal cord injury. For this reason, we analysed the subgroup of SCI individuals with a high probability of developing neuropathic pain (based on PD-Q classification) separately. When considering the participants from this group who completed the study, the main findings supporting efficacy are: 1) NFX88 significantly reduced VAS scores over time in the 2.1 g/day group (p = 0.019) and in the 4.2 g/day group (p = 0.042) after NFX88 treatment had begun; and 2) a more than 2-point reduction suggested this dose had the potential to have a large effect on pain.

As with all clinical trials, the limitations related to the study design must be considered. The exclusion criteria used, for example, limit our ability to generalize these findings to central neuropathic pain of aetiologies other than SCI. Also, the results of this 90-day trial may not extrapolate to longer periods of treatment. In our study, for practical and ethical reasons, pregabalin treatment and other neuroactive drugs were kept stable. Thus, future studies are required to establish its potential efficacy in combination with other analgesic drugs. Although the median pain score appeared to be improved at the end of the 3-month treatment period, a large percentage of SCI individuals did not experience a satisfactory reduction in pain (e.g. they were classified as non-responders). This is, however, not surprising because neuropathic pain due to SCI is often severe and difficult to treat [30]. Moreover, none of the treatments currently employed have resulted in a significant reduction in neuropathic pain in all treated people. In this context, it has been found that less than half of the individuals with SCI reported a reduction in pain after treatment with pregabalin or opiates [10, 31].

Overall, due to good safety and tolerability profiles, our findings make NFX88 an attractive therapeutic option, as a second-line treatment, for SCI-related pain, because many current options are limited by a lack of efficacy or the presence of severe side effects. Moreover, the observed effect (to be confirmed in further trials) in present trial was additional to that of pregabalin (administered to all participants in the present trial) and other compounds administered to manage pain, most likely due to their divergent mechanisms of action. The primary aim of this study was to compare the safety and to have a preliminary information about efficacy of three doses of NFX88 and to collect a sufficient amount of data on positive response rates to allow calculation of a suitable group size for a well-powered efficacy study.

## Data Availability

Data are available from Neurofix Pharma S.A. and the corresponding author on reasonable request.
